# Mitigating Contemporary Trauma Impacts Using Ancient Applications

**DOI:** 10.3389/fpsyg.2022.645397

**Published:** 2022-08-02

**Authors:** Gavin Morris, Rachel Groom, Emma Schuberg, Judy Atkinson, Caroline Atkinson, Miriam-Rose Ungunmerr-Baumann

**Affiliations:** ^1^College of Education, Charles Darwin University, Darwin, NT, Australia; ^2^Northern Institute, College of Indigenous Futures, Arts and Society, Charles Darwin University, Darwin, NT, Australia; ^3^We Al-li Pty Ltd, Goolmangar, NSW, Australia; ^4^Green River Aboriginal Corporation, Nauiyu Nambiyu, Daly River, NT, Australia

**Keywords:** trauma, Dadirri, COVID-19, indigenous practice, traditional healing

## Abstract

The COVID-19 pandemic represents the most significant global challenge in a generation. Based on extant data from previous pandemics, demographic, occupational, and psychological factors have been linked to distress and for some vulnerable members of society. COVID-19 has added to the layers of grief and distress of existing trauma. Evidence-based frameworks exist to guide our individual and collective response to reduce the trauma associated with the experience of a pandemic. Pandemic and post-pandemic measures to ameliorate impacts require a multi-disciplined approach, central to which is community connectedness, resilience, and access to support. We advocate for the acceptance and broader application of Dadirri, a healing practice held by the Ngan'gikurunggurr and Ngen'giwumirri Aboriginal people of the Daly River region in the Northern Territory, Australia. This modality engages therapeutic phases that are comparable with other practiced trauma therapies. The demonstrated therapeutic outcomes from Dadirri can be attained through an individualistic or in a relational engagement context. This practice is accessible to all ages, is non-specific to gender and is suitable for people constrained in their mobility or limited by resources, pertinent in pandemic affected settings.

## Introduction

The effects of a global pandemic can be profound on individuals and communities. Previous epidemics have induced widespread fear, loneliness, and psychological sequelae; COVID-19 has induced similar effects (Alradhawi et al., 2020). Empirical studies have shown that people who tested positive for COVID-19 had experienced poorer mental health outcomes. Individuals with a COVID-19 diagnosis have been found to have major psychological distress, anxiety, depression, and other mental health problems compared to people without infection (Guo et al., 2020; Hao et al., 2020; Huang and Zhao, 2020; Rogers, 2020; Hossain et al., 2020b). Psychological risk factors have been identified that predict for poorer mental health outcomes during the pandemic, these include psychological inflexibility (Li et al., 2020; Sultana et al., 2020), limited social support (Wang et al., 2020b), lack of cognitive control over emotions (Chirico et al., 2020), worry (Rogers, 2020; Hossain et al., 2020b), ineffective or maladaptive use of emotion regulation and coping strategies (Patel et al., 2018; Ehrlich et al., 2020; Pappa et al., 2020). Consequently, the lockdowns imposed throughout the pandemic, albeit crucial to reduce COVID-19 infection rates, have apparently exacerbated existing mental health issues. Social interaction is broadly linked with psychological health, social opportunities, and employment; thus, restricting these measures is evidenced to be profoundly distressing to those experiencing strict isolation.

Since the pandemic, research has explored the insulating factors that have supported the mental health of individuals impacted by the pandemic. Studies on psychological resilience, found a negative relationship between resilience and mental health problems such as depression, anxiety, and somatization (Goyal et al., 2020; Huang and Zhao, 2020; Walton et al., 2020) during the pandemic. Empirical studies demonstrate that greater social support, increased levels of mindfulness, cognitive control over emotions, and greater psychological flexibility, predicts the relationship between illness perceptions, resilience, and mental health problems (Chirico et al., 2020; Kavoor, 2020; Li et al., 2020; Mamun and Griffiths, 2020). This suggests that people who accessed more social support, and who were able to flexibly adapt their coping strategies in the event of distress, showed the most positive mental health outcomes during the pandemic (Hossain et al., 2020a). Additionally, research found social cohesion, measured in terms of sense of belonging, to be associated with mental wellbeing during lockdowns (Mamun and Ullah, 2020; Hossain et al., 2020a; Wang et al., 2020a). On this premise, we describe a culturally appropriate praxis that enables greater access by distressed peoples to increase their sense of belonging and connectedness during a pandemic (Atkinson et al., 2010; Morris, 2019).

## What Is Dadirri?

Dadirri is a healing framework of the Ngan'gikurunggurr and Ngen'giwumirri Aboriginal people of the Daly River region in the Northern Territory, Australia. Dadirri involves listening to the inner self (feelings, thoughts, reactions) and taking responsibility for internal responses (Ungunmerr-Baumann, 2002). It is the “self” that yearns for connection with all others and its environment, and that strives for spiritual reconnection with the cosmos (Atkinson, 2001; Delauney, 2013). Despite slight variations in meaning, it seems many (if not all) Aboriginal languages have words for listening, wanting to listen, needing to listen. In Australia there are more than 250 Indigenous languages including 800 dialects. Each language is specific to a particular place and people. For example, Bundjalung people have a similar word which is ninganah meaning “shush” with an emphasis on listening; in Wiradjuri language, the equivalent is Winhangadhurinya, a word describing meditation, deep listening, knowing, and reflecting.

The practice of Dadirri aims to lessen the effects of trauma and provide a pathway of empowerment and resilience for individuals and community. The diverse manifestations of trauma in this remote Aboriginal community necessitated an urgent and Indigenous-led response (Atkinson, 2002; Morris, 2019). Some commonalities experienced by the community align with the psychological distress observed during national emergencies, mostly stemming from uncertainty and fear, but also from the grief of separation, loss, and shame—a pain that is deep and unresolved. Similarly, where COVID-19 has revealed and exacerbated uncertainties and fears, Dadirri as a practice of listening and being seen during painful lockdowns and ongoing unresolved situations, can partially transcend separation by the sharing supported by online communities.

## Principles of Dadirri

**Figure 1:**
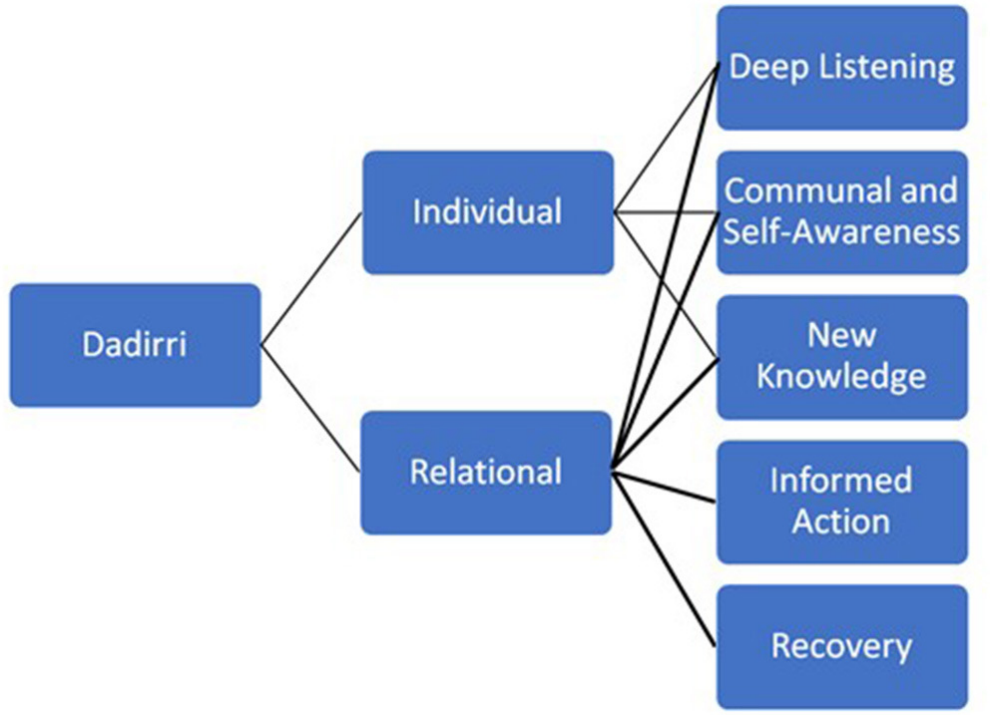
Therapeutic phases of Dadirri as applied through individualistic and relational pathways.

At its core, Dadirri enables a cyclical process of deep, heart-based listening, which witnesses, and empathizes *via* an individualistic or relational pathway (Figure 1). The great strength of Dadirri in this context is two-fold. Firstly, the cyclical, deep listening and reflection promoted through dialogue; Dadirri encourages relationships built on trust and respect, providing an opportunity to create the co-directional sharing of knowledge (Atkinson, 2002; West et al., 2012); that effectively attends to oppression and marginalization. The principle of reciprocity and connection are fundamental tenets to Dadirri and will shape the way stories and information are shared. Collectively, Dadirri is informed by the concept of community—all people matter, all people belong (Atkinson, 2001; Ungunmerr-Baumann, 2002; Morris, 2019). Secondly, it places recognition on community care and practice, valuing the contribution people make in their activities of relating, defining, and narrating their life experiences. There is a need to honor the integrity and fidelity of community in both its dynamic diversity and its interconnected unity (Atkinson, 2002).

## Healing Through Dadirri

The pandemic and the measures to mitigate it, have resulted in widespread restrictions placed on all aspects of people's everyday life. Attending to the pressures of society (e.g., social commitments, employment) is a daily concern for many and restricting people from leaving their home, for example, has caused great anguish and despair. In Dadirri, you are grounded in the present, time stands still. As Ngan'gikurunggurr Elders assert, “we are the river people—we cannot hurry the river, we need to move with the current and understand its ways.” During lockdown, people could no longer fill their life with “busyness” they were forced to stop, to be present, a scenario rarely experienced by many. Indeed, people yearn for this “busyness” as it distracts us from the silence, and the thoughts which can consume us while being grounded in the “present” (Morris, 2019).

## Comparison With Eastern and Western Approaches

Widely regarded in contemporary terms as “mindfulness”, a practice of deep awareness in the present moment and what is going on within and around oneself (Hanh, 1976) can be characterized by openness, curiosity, and acceptance (Bishop et al., 2004). There are multiple therapeutic modalities for trauma that incorporate techniques analogous to mindfulness as a critical first phase, these include Empowerment and Resilience Therapy, Acceptance and Commitment Therapy and Mindfulness-Based Cognitive Therapy. Chodron (2001) explained that there are four aspects that are fostered when people meditate: commitment, awareness, willingness to experience emotional distress, and attention to the present moment. Similarly, Dadirri integrates deep-listening and self-awareness, these phases are regarded as key for true connection and the dissolution of self-object dualism. Transformative learning theorists describe a state from which transformation is born. According to McWhinney and Markos (2003), that state is attained when one withdraws from the world to an inner place from which new insights and frames of reference emerge. This was observed as the “aha” moment in participants enduring the final stages of Dadirri practice: new knowledge, informed action, and recovery. Participants described a sense of overwhelming peace (Morris, 2019).

**Figure 2:**
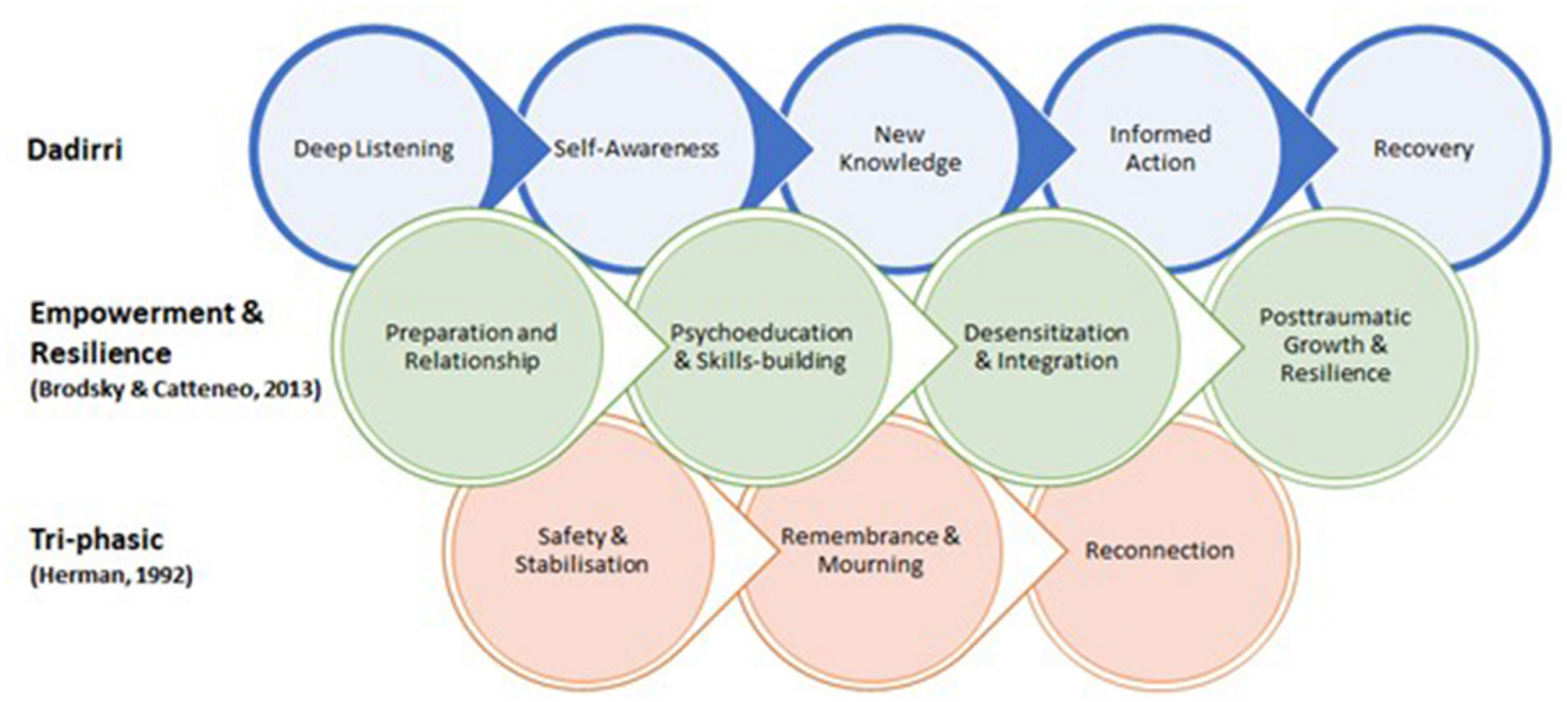
Comparative models of trauma treatment.

Dadirri as a practice has been used to endure painful emotions in ways that are not typical of Western approaches but broadly aligns with eastern Buddhist philosophies. While there are important similarities between Dadirri, Buddhist thought and some schools of Western psychology, there is value in recognizing their common objectives. The varied perspectives have parallel goals where they seek to foster growth, understanding, and freedom from suffering (Figure 2). Herman (1992) constructs a tri-phasic model that moves toward reconnection, while Brodsky and Cattaneo (2013) present stages of empowerment and resilience that build cumulatively. Dadirri aligns with these Western models of recovery such as in similar preparatory techniques, collective shifts during deep listening and self-awareness that supports the process to build toward re-integration of conscious knowing and doing. Furthermore, these varied perspectives share a fundamental assumption that there is an inherent potential within each person toward continual growth (Kumar, 2002). Practicing these behaviors as interventions in both pleasant and unpleasant circumstances, such as the COVID-19 pandemic and its after-effects, helps people consciously recognize themselves. This recognition of self is part of a greater context, not as good or bad, but as part of a changing universe and within a community.

## Dadirri as Healing From Trauma and Colonization

The importance of Dadirri as an Indigenous healing practice was articulated in a recent study on the impact of colonization in Daly River (Morris, 2019). Participants recognized the contemplative process of listening and learning from the stories of others and it allowed for healing to occur and increased their sense of community and connection (Morris, 2019). As illustrated in Figure 1, the therapeutic phases have both individual and relational aspects, and to fully appreciate Dadirri, one needs to move from the individualistic, to a collective perspective. By way of example, one participant stated that “it was this feeling of belonging and community which allows stories to be shared without fear of judgment”. Dadirri permeates every aspect of life, continually renewing the spirit, bringing peace and contemplation. As the recounts of trauma were repeated over time, another participant revealed “your story eventually changes, and the pain of trauma is released and replaced with love and acceptance as the healing process begins”.

Utilizing Dadirri within a local Nauiyu community context provides an opportunity for members to re-engage, trust and value their own cultural practices. Rather than a quick fix or the need to fix and change what arises, Dadirri asks of us to allow each moment, each breath, each story to unfold naturally. Giving us the opportunity to accept what is arising for us, or in us, at each successive moment of experience, without the need to fix, fight or avert what is arising. Empowering people through the learning and application of their traditional healing practices and knowledge is demonstrated to increase their individual and collective health, strength, and wellbeing (Atkinson et al., 2010; Barnabe, 2021).

For non-Indigenous people Dadirri allows engagement in a practice that has been shared across thousands of generations attributing equity to an accessible and beneficial therapy. Emerging research suggests that mainstream perceptions and subsequent policy implementations of Indigenous healing practices reflect the attitudes formed during the decline of Indigenous healing practice throughout colonization (Gone, 2013; Dudgeon et al., 2017). Dadirri offers a treatment option for practitioners in trauma therapy.

## Relevance to COVID-19 and Post-Pandemic Futures

The persistent trauma from COVID-19 requires a spectrum of resilience from individual and community-level acts to shift paradigms and policy, to combat the inequities of complex trauma through a foundation for collective healing. Public health challenges necessitate mobilizing resources available within and between contexts. In COVID-19, biopsychosocial challenges such as anxiety, fear and isolation experienced by individuals may be addressed by local and regional organizations through enhancing collaboration and extending social capital. Chen and Bonanno (2020) offer a spectrum of temporal elements of resilience that demonstrate multifactor social capital aspects contributing to resilience and recovery. Dadirri similarly draws on individual and collective community responses that support healing from trauma, such as in this case, COVID-19 effects. Supporting healing from COVID-19 adversity and building resilience by maintaining a sense of equilibrium can be achieved through connectedness to community and/or resilience resources; this is a key protective factor during times of hardship. Access to regular treatment and support during a pandemic is a major challenge but is necessary for limiting long-term consequences for mental health (Moreno et al., 2020; Pfefferbaum and North, 2020). Factors underlying the concept of Western mental health should also be addressed, such as expectations around lifestyle and sets of rigid strategies that prove insufficient in pandemic times. Dadirri is a flexible and accessible technique, demonstrated to be an appropriate and supportive treatment for people who have experienced trauma (Atkinson, 2002; Morris, 2019). Addressing the trauma in the current pandemic cultivates fertile ground for trauma-informed healing and recovery in our communities, beyond the COVID-19 aftermath.

## Trauma Aftermaths

The American Psychological Association (2016) defines trauma as events that pose a significant threat (physical, emotional, or psychological) to the safety of the victim or loved ones and are overwhelming and shocking. Current events, such as a pandemic, may aggravate past wounds for individuals who have experienced trauma in other areas of their life, requiring additional support measures. Persistent trauma that is not buffered by early intervention can permanently alter our DNA, promote vulnerability to future stressors (Youssef et al., 2018; Thumfart et al., 2022), and may be transmitted to future generations (Atkinson, 2002; Lavallée et al., 2015; Yehuda et al., 2016). As the challenges arising from the COVID-19 pandemic continue to evolve, so too have the significant and varying psychosocial demands on the global population (Ho et al., 2020; Xiang et al., 2020; Ammar et al., 2020); Psychosocial shifts can occur due to confinement, economic shutdown, financial pressure, and social distancing.

With access to psychosocial services being restricted within the pandemic, alternative frameworks for healing justify consideration. Within a community, an individual can take steps to disrupt negative responses to trauma and community collective action can also be enabled. Successful community-based programs for the treatment of trauma are implemented in diverse contexts throughout the world (Ertl et al., 2011; Konanur et al., 2015; López-Zerón and Parra-Cardona, 2015).

## Intersections of Resilience

COVID-19 has impacted the population in an unequal manner. Haase (2020) highlights the inequality and justice challenges arising from the COVID-19 crisis and how exposure and vulnerability to COVID-19 emerges at the intersection between different dimensions of disadvantage and marginalization. The degree to which the pandemic impacts a place and exposes its people to health risk is without doubt related to the socio-spatial inequalities and socio-economic status or lack thereof (Simon, 2020; Haase, 2020) resilience. As was the case in previous pandemics, the trauma associated with COVID-19 has disproportionately impacted vulnerable population groups, particularly those who have experienced past trauma and have pre-existing differences in health status (Gaynor and Wilson, 2020).

## Recommendations and Future Directions

Dadirri works by influencing behavioral change through developing deep acceptance and prompting holistic behavior. It is integral to the community healing program in several remote communities in Australia with complex trauma. The healing capacity of Dadirri has been successfully applied in a range of national and international contexts. It is a profoundly important healing practice to the Nauiyu community and whilst it is important to acknowledge the place-based significance of Dadirri, hundreds of people from across the world have traveled to Nauiyu to practice Dadirri over many years. There are an increasing number of evidence-based programs that have used Dadirri “off Country” to establish respectful healing environments to address symptoms of trauma (Bevis et al., 2020; Coombes et al., 2020).

Although studies are still emerging from the COVID-19 pandemic, Dadirri as an ancient application that can be transferred into multimodal contexts may be compared with similar holistic practices studied during the pandemic, such as yoga and mindfulness-based online interventions (Godara et al., 2021; Verma et al., 2021). Dadirri has also been applied to broader organizational and service-level systems of care to build community healing networks through educational settings, primary health care providers and other government and non-government organizations (Bevis et al., 2020; Woodland, 2021). Training in Dadirri offers professionals and service providers a way of engaging and responding to clients who are triggered by authority figures and procedures that trigger trauma. As psychological, medical, and community health practitioners continue to deal with the aftermath of COVID-19 procedures and authority figures, Dadirri may be applied to create safe healing spaces. Developing a posture of awareness and deep listening through Dadirri counters the dismissive and insensitive “expert” and instead creates gentle and co-invitational healing spaces. Dadirri is adaptable in a range of social contexts, with its therapeutic capacity proven in solitary settings, small groups in face-to-face environments and devices with online platforms (Sunderland et al., 2020) which also reduce feelings of isolation for affected patients, their families and members of the public (Xiang et al., 2020).

The importance of Dadirri as part of a broader body of Indigenous healing practices has been recognized as significant by national peak-body mental health organizations including, the Psychotherapy and Counseling Federation of Australia (PACFA^1^)- the national peak body for counselors and psychotherapists and for professional associations in the counseling and psychotherapy field in Australia. PACFA has recently launched an Indigenous led college within its organization to accredit Indigenous Healing Practices and training standards. The College of Aboriginal and Torres Strait Healing Practices (CATSIHP) has developed training standards which encompass “traditional contemporary, and emerging healing modalities created by and founded in Indigenous wisdom, systems of knowledge and ways of being” (https://www.pacfa.org.au/). The association acknowledges Indigenous healing practices in its constitution and is working to accredit and validate Indigenous healing practices, such as Dadirri, as equal with traditional Western and Eurocentric counterparts. Indeed, such has been the emergence of Indigenous Healing practices in Western health settings, CATSIHP has been approached by large Australian private health insurers to explore the potential of including traditional healing practices in their mainstream insurance packages.

The uncertainty of the COVID-19 pandemic; the confinement, isolation, physical distancing, and other confinement strategies which have resulted in societal breakdown and economic collapse has seen a profound decline in mental health. Trauma is a multisensory experience; therefore, we need multisensory interventions—whole of body interventions which not only involve the individual but also the collective. Dadirri is an adaptable Indigenous healing practice, proven in a range of contexts to offer therapeutic responses to trauma, healing the wounds of separation and restoring our living flow.

## Data Availability Statement

The raw data supporting the conclusions of this article will be made available by the authors, without undue reservation.

## Ethics Statement

The studies involving human participants were reviewed and approved by Charles Darwin University Ethics Committee. The patients/participants provided their written informed consent to participate in this study.

## Author Contributions

GM, RG, ES, JA, CA, and M-RU-B wrote the manuscript. GM gathered and analyzed the data. All authors have read and approved the final manuscript.

## Conflict of Interest

CA was employed by the company We Al-li Pty Ltd. Author Emeritus Professor JA is an advisor for the company We Al-li Pty Ltd without any financial interests in this company. The remaining authors declare that the research was conducted in the absence of any commercial or financial relationships that could be construed as a potential conflict of interest.

## Publisher's Note

All claims expressed in this article are solely those of the authors and do not necessarily represent those of their affiliated organizations, or those of the publisher, the editors and the reviewers. Any product that may be evaluated in this article, or claim that may be made by its manufacturer, is not guaranteed or endorsed by the publisher.

## Footnotes

^1^Psychotherapy and Counselling Federation of Australia. Available online at: https://www.pacfa.org.au/ (accessed March 04, 2022).
